# Risk Assessment Using the Association Between Renin-Angiotensin Genes Polymorphisms and Coronary Artery Disease

**DOI:** 10.7759/cureus.14083

**Published:** 2021-03-24

**Authors:** Mohamed Riad, Prakash Adhikari, Sanket Bhattarai, Ashish Gupta, Eiman Ali, Moeez Ali, Jihan A Mostafa

**Affiliations:** 1 Internal Medicine, California Institute of Behavioral Neurosciences & Psychology, Fairfield, USA; 2 Internal Medicine, Piedmont Athens Regional Medical Center, Athens, USA; 3 Research, California Institute of Behavioral Neurosciences & Psychology, Fairfield, USA; 4 Psychiatry, California Institute of Behavioral Neurosciences & Psychology, Fairfield, USA

**Keywords:** ace polymorphism and myocardial infarction, ras polymorphism and coronary artery disease, ras polymorphism and hypertension, enos polymorphism and coronary artery disease

## Abstract

Coronary artery disease (CAD) is a multifactorial disease that involves genetic and environmental interaction. In addition to the well-known CAD risk factors, such as diabetes mellitus, hypertension, hyperlipidemia, and atherosclerosis, it has a genetic component that predisposes to its occurrence even in young people. One of the most commonly studied genes that increase the susceptibility to CAD is renin-angiotensin system (RAS) genes polymorphisms mainly angiotensin-converting enzyme gene (ACE) polymorphisms, angiotensinogen polymorphisms, angiotensin- II type 1 receptor gene polymorphisms, and many other genes. These genetic polymorphisms have a direct association with CAD development or indirect association through causing atherosclerosis and hypertension which, in turn, are complicated by CAD later on. The difference between genetic mutations and polymorphisms lies in the frequency of the abnormal genotype. If the frequency is 1% and more in the general population, it is called polymorphism and if it is less than 1%, then it is called a mutation.

According to our findings, after thorough searching, which support the association of RAS genes polymorphisms with premature CAD, hypertension, hypertrophic cardiomyopathy, and atherosclerosis, we recommend additional studies in the form of clinical trials and meta-analyses aiming to create a specific diagnostic tool for CAD risk assessment and discovering the high-risk people as early as possible. Targeted gene therapy, being the future of medicine, needs to be taken into researchers' consideration. It can have promising results in these cases.

## Introduction and background

Cardiovascular diseases are the leading cause of death and are considered major causes of morbidity and mortality worldwide. Centers for Disease Control and Prevention (CDC) reported that, in the United States, one of every four deaths is due to cardiovascular diseases, and every 36 seconds pass, a person passes away as a result of their diseased heart. Among all cardiovascular diseases, coronary artery disease (CAD) is the most common, which affects approximately 18.2 million adults above 20 years old, with a prevalence of about 6.7%. Interestingly, CAD is not exclusive to older adults, but it also affects young people (premature coronary artery disease), and that supports the idea of genetic involvement in the pathogenesis of CAD and its environmental interaction [1,2].

CAD is a multifactorial disease triggered by environmental as well as genetic factors [3]. Not only smoking, diabetes mellitus, obesity, hypertension, and low-density lipoprotein (LDL) increases the risk of CAD, but also genetic susceptibility plays a role. Several genetic polymorphisms were studied for their association with CAD and myocardial infarction (MI) risk, such as factor v Leiden, factor II prothrombin, endothelial nitric oxide synthase (eNOS), methyltetrahydrofolate reductase (MTHFR), plasminogen activator inhibitor type 1 (PAI-1), paraoxonase (PON-1), APOA5 (apo-lipoprotein A5) [4-8]. Moreover, apolipoprotein-E (apo-E) polymorphism is considered an independent predictor of CAD at a young age [9]. However, the most studied genes are renin-angiotensin system (RAS) gene polymorphisms which are involved in hypertension, left ventricular hypertrophy, hypertrophic cardiomyopathy, and CAD [10-13].

Angiotensin-converting enzyme (ACE) converts angiotensin-1 (AT-I) to angiotensin-2 (AT-II) which increases blood pressure by causing vasoconstriction, increases aldosterone secretion from adrenal glands, stimulates the sympathetic nervous system, induces the growth, cell migration, and mitosis of smooth muscles, increases the synthesis of collagen in fibroblasts leading to thickening of the vascular wall, myocardium, and fibrosis [14], so it is involved in cardiac remodeling [15]. AT-II is also pro-inflammatory through inducing the production of tumor necrosis factor-α and interleukin-6; thus, it plays a role in atherosclerotic plaque formation. ACE polymorphism leads to increased synthesis and activity of AT-II. Also, ACE causes degradation of bradykinin which also affects the cardiovascular functions as a vasodilator [16].

Insertion/deletion (I/D) polymorphism of the ACE gene has been the most frequently studied one. ACE gene is present on chromosome17q23 and consists of 25 introns and 26 exons [17]. Insertion or deletion of a 287 base pair repeat sequence in the 16th intron is associated with an increase in circulating ACE as well as its level and activity on the heart tissue [16,18,19]. Furthermore, some variants of angiotensin II type 1 receptor (AT1R) and angiotensinogen (AGT) have also shown some association. AT1R 1166A/C polymorphism is associated with CAD and MI especially if combined with ACE polymorphism and smoking [20-22]. AGT MM and AT1R CC genotypes act synergistically with ACE DD genotypes to increase the risk of CAD [21,23-26]. Moreover, it has been shown that DD genotype increases the risk of MI in young patients [27], recurrent MI and post-MI complications in addition to some behavioral traits particularly type A personality [28].

Before we delve into our topic, a question may come to mind. Is there a difference between genetic polymorphism and mutation? The answer is yes. Then what is that difference? The answer is "how frequent is it?". Both polymorphism and mutation represent a change in the DNA sequence away from normal. However, to be labeled as polymorphism, the abnormal allele should have a frequency of 1% or more, in contrast to the mutation in which the abnormal genotype occurs at a frequency of less than 1% in the general population.

The main aim of our review study is to discuss the association between RAS gene polymorphisms and the risk of CAD, either directly or through influencing other risk factors such as hypertension, hyperlipidemia, and atherosclerosis or through interacting with other genetic polymorphisms.

## Review

Not only RAS gene polymorphisms are considered an independent risk factor of developing CAD, but they can also influence other CAD risk factors such as hypertension, atherosclerosis, and thrombosis. The net result is a higher risk of developing CAD in individuals carrying these polymorphisms.

RAS genes polymorphisms predict the risk of CAD and MI

ACE gene polymorphism is considered a strong independent risk factor for CAD and MI especially in a low-risk population such as non-smokers, non-diabetics, and normotensive people [14]. A low HDL level can potentiate the risk [29]. The DD genotype frequency is found to be high in MI patients and their first-degree relatives in addition to patients with hyperlipidemia [22,30,31]. AT1R C allele is associated with an increased risk of MI in the general population [20,21]. The I/D ACE gene polymorphism results in increasing ACE level which converts AT-I to AT-II leading to an increased AT-II level. AT-II, in turn, causes vasoconstriction, aldosterone secretion from the adrenal cortex, sodium retention by kidneys, prothrombotic activity through stimulating PAI-1 and platelet aggregation, sympathetic stimulation, releasing the inflammatory mediators as well as cardiac remodeling, and that explains the mechanism underlying the association of I/D polymorphism with cardiovascular problems [4,19]. Consequently, the importance of screening for genetic risk factors should be taken into consideration [26]. Given the above, RAS gene polymorphisms are considered a culprit of CAD and MI as they play a major role in their pathogenesis, and their presence in any individual means that they are at a higher risk of developing adverse cardiac events.

On the other hand, a study showed that there is no significant impact for ACE I/D gene polymorphism on the risk of MI by comparing 684 patients with acute MI in CCU in two centers with 537 control subjects from the base population [32]. Another study concluded that the adjusted relative risk of the D allele was 1.07 for ischemic heart disease and 1.05 for myocardial infarction, so the ACE genotype does not significantly increase the risk of ischemic heart diseases (IHD) or MI [33]. These contradictory results may be attributed to the low sample size.

RAS genes polymorphisms and premature coronary artery disease

It is well-known that CAD is a disease of older adults with risk factors like hypertension, hyperlipidemia, smoking, or diabetes mellitus; however, studies have shown that ACE DD, ACE ID, and AGT MM genotypes are considered independent risk factors of premature CAD [24,25]. Additionally, AT1R and AT2R gene polymorphisms are also found to increase the susceptibility of Egyptian people to premature CAD as well as metabolic syndrome [1,2]. Moreover, the synergistic interaction between RAS gene polymorphisms and other metabolic risk factors is greatly involved in the risk of premature MI [27]. Also, apo E polymorphism is involved in the pathogenesis of accelerated CAD at a younger age. In light of the above, these high-quality studies with a larger sample size are showing that RAS gene polymorphisms can lead to premature CAD even without other metabolic risk factors, so we can see that, unfortunately, no age is immune against CAD and MI.

On the contrary, a study showed that there is no association between ACE genotypes and premature CAD [34], but that could also be due to the low sample size, as the study sample included only 201 patients with premature MI and 140 age and sex-matched healthy individuals. If done on a larger number of patients, it may yield different results. The studies supporting the idea of the association between RAS genes polymorphisms and premature CAD and MI are including a larger sample size and more evidence-based, so they are more likely to be right.

RAS genes polymorphisms and hypertension/left ventricular hypertrophy/hypertrophic cardiomyopathy

RAS gene polymorphisms are not confined to their association with CAD and MI. They are also involved in the pathogenesis of arterial hypertension (AH) as well as hypertrophic cardiomyopathy (HCM) and they can contribute to the occurrence of clinical manifestations and complications associated with AH and HCM [12]. As mentioned before, the mechanism by which ACE gene polymorphism causes adverse cardiovascular events is based on the action of AT-II. AT-II level and activity increases as a result of ACE gene polymorphism, then AT-II interacts with type 1 receptors to exert the following actions: vasoconstriction, sympathetic stimulation, increasing aldosterone synthesis and secretion from the adrenal cortex, stimulating the growth and mitosis of vascular smooth muscle cells as well as fibroblasts proliferation with increasing collagen synthesis. The net effect of these actions is vascular wall thickening, myocardial thickening, and fibrosis [35]. That is how AT-II is a culprit of AH and vascular wall damage and left ventricular hypertrophy (LVH) associated with hypertension. Logically, blocking AT-II type 1 receptors by angiotensin receptor blockers (ARBs) such as losartan or valsartan will exert a protective role against these adverse cardiac events. That is why ARBs drugs are prescribed for patients who are discharged after MI to prevent the cardiac remodeling and fibrosis of the myocardium post-MI which is mediated by A-II as mentioned.

Also, ACE I/I and D/D genotypes were more frequent in the hypertensive patients compared to a control healthy group emphasizing the significant association between ACE gene polymorphisms and early-onset essential hypertension in people less than 40 years old [10,11]. Furthermore, ACE I/D polymorphism is associated with HCM according to a systematic review and meta-analysis of case-control studies [13] which are of high quality and more evidence-based, and have less chance of bias.

A study showed that A-240T, T-93C, and A2350G are associated with decreased serum ACE and diastolic blood pressure [36]. However, 104 individuals only were included in this study, so the results can be controversial. RAS genes polymorphisms play a major role in the pathogenesis and complications of essential hypertension, LVH, HCM, and cardiac remodeling; thus, highlighting the beneficial effect of angiotensin-converting enzyme inhibitors (ACEIs) such as enalapril and angiotensin receptor blockers (ARBs) such as losartan in these conditions as well as protecting the kidneys.

RAS genes polymorphisms and atherosclerosis

Atherosclerosis is a multifactorial process that begins at an earlier age and remains sub-clinical for many years before the clinical manifestations develop. Hyperlipidemia, diabetes mellitus, smoking, and hypertension are major risk factors; however, the genetic component of this problem should not be ignored since genetic susceptibility can accelerate the pathophysiology of atherosclerosis and its clinical outcomes even with controlling the other metabolic risk factors. The atherosclerosis process can be divided into four main steps: (1) endothelial dysfunction - that is the first step in atherosclerotic plaque formation; (2) lipoprotein and cholesterol deposition - after endothelium disruption, LDL molecules enter the vessel wall, get oxidized by free radicals then get engulfed by macrophages forming foam cells and fatty streaks; (3) inflammatory reaction - the oxidized LDL and the injured endothelium release several inflammatory mediators and attract inflammatory cells like macrophages into the vessel wall; (4) smooth muscle cells and fibrous cap formation.

The most important step and the main trigger of atherosclerotic plaque formation is LDL oxidation and this is where AT-II comes in. AT-II is pro-atherogenic through induction of aggregation of the native and oxidized LDL into the vessel wall [37]. Another clinical trial demonstrated that the ACE DD genotype increases the risk of carotid artery atherosclerosis only; however, the ACE I/D genotype increases the risk of MI, HCM, LVH as well as carotid atherosclerosis [38]. ACE D/-240 T holotype can predict the occurrence of peripheral arterial disease (PAD) and its complications in the long run and that has been shown by analyzing 281 patients with PAD and 485 control group of the same age and sex and other risk factors [39].

Other genes that can increase the susceptibility to CAD and MI

CAD is a complex and multi-factorial disease that includes multiple genes as well as genetic-environmental interaction. We previously discussed how ACE polymorphisms can lead to CAD by increasing the level and activity of A-II. However, that is not the only effect of ACE genotypes. ACE DD genotype can influence the fibrinolysis pathway which is involved in thrombus formation and degradation. ACE DD genotype is found to increase the serum level of plasminogen activator inhibitor-1 (PAI-1) by inducing its synthesis, therefore accelerates the atherosclerosis process [5]. PAI-1 is a protease inhibitor whose main function is to inhibit tissue plasminogen activator (tPA) and urokinase; thus, suppresses the fibrinolysis and puts the individual at a greater risk of thrombus formation and accelerated atherosclerosis.

Paraoxonase-1 (PON-1) is another gene involved in CAD. Two studies, one of them is conducted on 90 patients with CAD compared to a control group of 90 healthy individuals, and the second one is conducted on 414 patients undergoing their first coronary angiography and comparing them with another control group with no CAD or risk factors found that there is a higher frequency of PON-1 genotype in patients with CAD (PON-1 Arg 192 in the second study) suggesting that PON-1 genotypes can be an independent risk factor of CAD and three-vessel stenosis; however, this study is performed on a small number of patients and a larger sample size may be necessary to confirm this association [6,7]. Another study demonstrated that the frequency of PON A/G 573 genotype was higher among the patients with CAD, and the interaction with ACE increases the risk of CAD [8]. So, that means that some PON-1 genotypes are associated with the development of CAD.

Endothelial nitric oxide synthase (eNOS) is also involved in the pathogenesis of CAD and the endothelial nitric oxide serum level is affected by ACE polymorphism. One of the main functions of ACE is the degradation of bradykinin, which exerts a vasodilator action by inducing the synthesis and release of vasodilator nitric oxide from the endothelium. Some polymorphisms in eNOS were recorded. One of them is a missense single-nucleotide change of G to T at position 894 (Glu298Asp) in exon 7 of the gene [4]. This polymorphism leads to decreased synthesis and activity of eNOS and nitric oxide; consequently, it is considered a risk factor for developing CAD [40].

At the end of our discussion, as it has been previously illustrated, RAS gene polymorphisms are a culprit of several cardiovascular diseases, some of them can be fatal or at least negatively affect the patient's quality of life at an earlier age than expected. So how can we interfere with these genetic polymorphisms to save those people with genetic predisposition from suffering serious cardiovascular events? And that is where the role of targeted gene therapy comes into play.

Limitations

We used only studies published in the last 10 years and studies that are published in the English language. We did not use quality check/assessment.

## Conclusions

Understanding the risks, pathogenesis, and causes of CAD has obviously changed recently. Nowadays, CAD is not restricted to a certain sex, age group, or risk factor. The occurrence of premature CAD and MI in young healthy persons without any known risk factor has redirected researchers' thoughts to another way looking for another hidden cause and eventually discovered that it is genetics and the heart. Genetic susceptibility is a key element in the multifactorial pathogenesis of CAD that should be taken into consideration. RAS plays a major role in cardiovascular hemodynamics. It makes sense that RAS gene polymorphisms are proven by high-quality studies to be associated with many cardiovascular problems mainly CAD. Some RAS gene polymorphisms are considered a predictive method of the individual's risk or susceptibility to developing CAD later. Other genotypes are linked to premature CAD, hypertension, hypertrophic cardiomyopathy, or atherosclerosis.

The association between RAS genes polymorphisms and CAD can have great clinical implications either on determining the individual's risk as early as possible so that we can interfere at the right time and prevent that progression, diagnosis, or even treatment of CAD. That will also have a great socio-economic impact and save many lives that were lost in the past without knowing what is wrong and their only problem was "bad genetics". Now we know what is wrong. We know where the problem is. So further studies in the form of clinical trials and meta-analyses are required to formulate a specific diagnostic tool based on the previously discussed considerations and there may be some extension beyond the diagnosis to the targeted gene therapy for high-risk individuals. If this comes into reality, it will do humanity an unforgettable favor.

## References

[1] Abd El-Aziz TA, Mohamed RH, Rezk NA (2014). Association of angiotensin II type I and type II receptor genes polymorphisms with the presence of premature coronary disease and metabolic syndrome. Mol Biol Rep.

[2] Abd El-Aziz TA, Hussein YM, Mohamed RH, Shalaby SM (2012). Renin-angiotensin system genes polymorphism in Egyptians with premature coronary artery disease. Gene.

[3] Mehri S, Mahjoub S, Finsterer J, Zaroui A, Mechmeche R, Baudin B, Hammami M (2011). The CC genotype of the angiotensin II type I receptor gene independently associates with acute myocardial infarction in a Tunisian population. J Renin Angiotensin Aldosterone Syst.

[4] Mokretar K, Velinov H, Postadzhiyan A, Apostolova M (2016). Association of polymorphisms in endothelial nitric oxide synthesis and renin-angiotensin-aldosterone system with developing of coronary artery disease in Bulgarian patients. Genet Test Mol Biomark.

[5] Kim Duk-Kyung, Kim Jong-Won, Kim Seonwoo (1997). Polymorphism of angiotensin converting enzyme gene is associated with circulating levels of plasminogen activator inhibitor-1. Arterioscler Thromb Vasc Biol.

[6] Vaisi-Raygani A, Ghaneialvar H, Rahimi Z (2011). Paraoxonase Arg 192 allele is an independent risk factor for three-vessel stenosis of coronary artery disease. Mol Biol Rep.

[7] Agirbasli M, Guney A, Ozturhan H (2011). Multifactor dimensionality reduction analysis of MTHFR, PAI-1, ACE, PON1, and eNOS gene polymorphisms in patients with early onset coronary artery disease. Eur J Cardiovasc Prev Rehabil.

[8] Pandey U, Kumari R, Nath B (2011). Association of angiotensin-converting enzyme, methylene tetrahydrofolate reductase and paraoxonase gene polymorphism and coronary artery disease in an Indian population. Cardiol J.

[9] Brscic E, Bergerone S, Gagnor A (2000). Acute myocardial infarction in young adults: prognostic role of angiotensin-converting enzyme, angiotensin II type I receptor, apolipoprotein E, endothelial constitutive nitric oxide synthase, and glycoprotein IIIa genetic polymorphisms at medium-term follow-up. Am Heart J.

[10] Ismail M, Akhtar N, Nasir M, Firasat S, Ayub Q, Khaliq S (2004). Association between the angiotensin-converting enzyme gene insertion/deletion polymorphism and essential hypertension in young Pakistani patients. BMB Rep.

[11] Moiseev VS, Demurov LM, Kobalava ZD (1997). The polymorphism of the angiotensin-converting enzyme gene in patients with hypertension, left ventricular hypertrophy and the development of a myocardial infarct at a young age. Preliminary report. [Article in Russian]. Ter Arkh.

[12] Stepanov VA, Puzyrev KV, Spiridonova MG, Pavliukova EN, Puzyrev VP, Karpov RS (1998). Polymorphism of angiotensin-converting enzyme and endothelial nitric oxide synthase genes in people with arterial hypertension, left ventricular hypertrophy, and hypertrophic cardiomyopathy. [Article in Russian]. Genetika.

[13] Luo R, Li X, Wang Y (2013). The influence of Angiotensin converting enzyme and angiotensinogen gene polymorphisms on hypertrophic cardiomyopathy. PloS One.

[14] Mata-Balaguer T, de la Herrán R, Ruiz-Rejón C, Ruiz-Rejón M, Garrido-Ramos MA, Ruiz-Rejón F (2004). Angiotensin-converting enzyme and p22phox polymorphisms and the risk of coronary heart disease in a low-risk Spanish population. Int J Cardiol.

[15] Albuquerque FN de, Brandão AA, Silva DA da (2014). Angiotensin-converting enzyme genetic polymorphism: its impact on cardiac remodeling. Arq Bras Cardiol.

[16] Cambien F, Costerousse O, Tiret L (1994). Plasma level and gene polymorphism of angiotensin-converting enzyme in relation to myocardial infarction. Circulation.

[17] Hmimech W, Idrissi HH, Diakite B (2017). Impact of I/D polymorphism of angiotensin-converting enzyme (ACE) gene on myocardial infarction susceptibility among young Moroccan patients. BMC Res Notes.

[18] Mehri S, Baudin B, Mahjoub S (2010). Angiotensin-converting enzyme insertion/deletion gene polymorphism in a Tunisian healthy and acute myocardial infarction population. Genet Test Mol Biomark.

[19] Danser AHJ, Schalekamp MADH, Bax WA, van den Brink AM, Saxena PR, Riegger GAJ, Schunkert H (1995). Angiotensin-converting enzyme in the human heart. Circulation.

[20] Feng X, Zheng BS, Shi JJ, Qian J, He W, Zhou HF (2014). A systematic review and meta-analysis of the association between angiotensin II type 1 receptor A1166C gene polymorphism and myocardial infarction susceptibility. J Renin Angiotensin Aldosterone Syst.

[21] Kaur R, Das R, Ahluwalia J, Kumar RM, Talwar KK (2012). Synergistic effect of angiotensin II type-1 receptor 1166A/C with angiotensin-converting enzyme polymorphism on risk of acute myocardial infarction in north Indians. J Renin Angiotensin Aldosterone Syst.

[22] Buraczyńska M, Pijanowski Z, Spasiewicz D, Nowicka T, Sodolski T, Czekajska TW, Ksiazek A (2003). Renin-angiotensin system gene polymorphisms: assessment of the risk of coronary heart disease. Kardiol Pol.

[23] Zhang Y, Yang T, Zhou W, Huang Y (2019). A meta-analysis on the association of genetic polymorphism of the angiotensin-converting enzyme and coronary artery disease in the chinese population. Rev Assoc Médica Bras.

[24] Vaisi-Raygani A, Ghaneialvar H, Rahimi Z (2010). The angiotensin converting enzyme D allele is an independent risk factor for early onset coronary artery disease. Clin Biochem.

[25] Sekuri C, Cam FS, Ercan E (2005). Renin-angiotensin system gene polymorphisms and premature coronary heart disease. J Renin Angiotensin Aldosterone Syst.

[26] Fatini C, Abbate R, Pepe G (2000). Searching for a better assessment of the individual coronary risk profile. The role of angiotensin-converting enzyme, angiotensin II type 1 receptor and angiotensinogen gene polymorphisms. Eur Heart J.

[27] Petrovič D, Zorc M, Kanič V, Peterlin B (2001). Interaction between gene polymorphisms of renin-angiotensin system and metabolic risk factors in premature myocardial infarction. Angiology.

[28] Malygina NA, Kostomarova IV, Vodolagina NN, Melent’ev IA, Melent’ev AS (2011). The genes of atherosclerosis and cardiovascular diseases. [Article in Russian]. Klin Med (Mosk).

[29] Baruah S, Chaliha MS, Borah PK, Rajkakati R, Borua PK, Mahanta J (2016). Insertion/insertion genotype of angiotensin i-converting-enzyme gene predicts risk of myocardial infarction in North East India. Biochem Genet.

[30] Reddy BP, Babu BMS, Karunakar KV (2010). Angiotensin-converting enzyme gene variant and its levels: risk factors for myocardial infarction in a South Indian population. Singapore Med J.

[31] Moradzadegan A, Vaisi-Raygani A, Nikzamir A, Rahimi Z (2015). Angiotensin converting enzyme insertion/deletion (I/D) (rs4646994) and Vegf polymorphism (+405G/C; rs2010963) in type II diabetic patients: association with the risk of coronary artery disease. J Renin Angiotensin Aldosterone Syst.

[32] Samani NJ, O’Toole L, Martin D (1996). Insertion/deletion polymorphism in the angiotensin-converting enzyme gene and risk of and prognosis after myocardial infarction. J Am Coll Cardiol.

[33] Lindpaintner K, Pfeffer MA, Kreutz R (1995). A prospective evaluation of an angiotensin-converting-enzyme gene polymorphism and the risk of ischemic heart disease. N Engl J Med.

[34] Rallidis LS, Gialeraki A, Varounis C (2009). Lack of association of angiotensin-converting enzyme insertion/deletion polymorphism and myocardial infarction at very young ages. Biomarkers.

[35] Fyhrquist F, Metsärinne K, Tikkanen I (1995). Role of angiotensin II in blood pressure regulation and in the pathophysiology of cardiovascular disorders. J Hum Hypertens.

[36] Firouzabadi N, Tajik N, Shafiei M, Ebrahimi SA, Bakhshandeh H (2011). Interaction of A-240T and A2350G related genotypes of angiotensin-converting enzyme (ACE) is associated with decreased serum ACE activity and blood pressure in a healthy Iranian population. Eur J Pharmacol.

[37] Nouryazdan N, Adibhesami G, Birjandi M, Heydari R, Yalameha B, Shahsavari G (2019). Study of angiotensin-converting enzyme insertion/deletion polymorphism, enzyme activity and oxidized low density lipoprotein in Western Iranians with atherosclerosis: a case-control study. BMC Cardiovasc Disord.

[38] Jeng JR (2000). Carotid thickening, cardiac hypertrophy, and angiotensin converting enzyme gene polymorphism in patients with hypertension. Am J Hypertens.

[39] Fatini C, Sticchi E, Sofi F (2009). Multilocus analysis in candidate genes ACE, AGT, and AGTR1 and predisposition to peripheral arterial disease: role of ACE D/-240T haplotype. J Vasc Surg.

[40] Teralı K, Ergören MÇ (2019). The contribution of NOS3 variants to coronary artery disease: a combined genetic epidemiology and computational biochemistry perspective. Int J Biol Macromol.

